# Assessing insurance claims as a measure for outpatient antimicrobial stewardship

**DOI:** 10.1017/ash.2025.10042

**Published:** 2025-06-30

**Authors:** Mariana M. Lanata, Jacob T. Kilgore, Brandi Holthaus, Jonathan M. Willis, Tess Anderson, Borden Samples, Jennifer Sparks, Joseph E. Evans, Bethany A. Wattles, Michael J. Smith

**Affiliations:** 1 Department of Pediatrics, Division of Pediatric Infectious Diseases, Marshall University Joan C. Edwards School of Medicine, Huntington, WV, USA; 2 Hoops Family Children’s Hospital, Huntington, WV, USA; 3 Department of Pediatrics, Division of Pediatric Infectious Diseases, Duke University, Durham, NC, USA; 4 Department of Information Technology, Marshall University Joan C Edwards School of Medicine, Huntington, WV, USA; 5 Marshall Health Network, Huntington, WV, USA; 6 Marshall University School of Pharmacy, Huntington, WV, USA; 7 Department of Pediatrics, Marshall University Joan C. Edwards School of Medicine, Huntington, WV, USA; 8 Independent Contractor, St. Louis, MO, USA; 9 Duke Center for Antimicrobial Stewardship and Infection Prevention, Durham, NC, USA

## Abstract

**Objective:**

This study evaluated Medicaid claims (MC) data as a valid source for outpatient antimicrobial stewardship programs (ASPs) by comparing it to electronic medical record (EMR) data from a single academic center.

**Methods:**

This retrospective study compared pediatric patients’ MC data with EMR data from the Marshall Health Network (MHN). Claims were matched to EMR records based on patient Medicaid ID, service date, and provider NPI number. Demographics, antibiotic choice, diagnosis appropriateness, and guideline concordance were assessed across both data sources.

**Setting:**

The study was conducted within the MHN, involving multiple pediatric and family medicine outpatient practices in West Virginia, USA.

**Patients:**

Pediatric patients receiving care within MHN with Medicaid coverage.

**Results:**

MC and EMR data showed >90% agreement in antibiotic choice, gender, and date of service. Discrepancies were observed in diagnoses, especially for visits with multiple infectious diagnoses. MC data demonstrated similar accuracy to EMR data in identifying inappropriate prescriptions and assessing guideline concordance. Additionally, MC data provided timely information, enhancing the feasibility of impactful outpatient ASP interventions.

**Conclusion:**

MC data is a valid and timely resource for outpatient ASP interventions. Insurance providers should be leveraged as key partners to support large-scale outpatient stewardship efforts.

## Introduction

It is estimated that 60%–95% of antibiotic prescriptions in the U.S. occur in the outpatient setting.^1^ Approximately 50% of antibiotic prescriptions are inappropriate, with 30% deemed unnecessary.^2^ A recent study in Tennessee found that only 31.4% of outpatient prescriptions were optimal in both antibiotic choice and duration of therapy.^3^ These findings highlight a substantial opportunity for improvement in outpatient antibiotic prescribing practices. To successfully reduce antibiotic overprescribing, it is crucial to develop effective outpatient stewardship interventions. Tracking and reporting is one of the core elements of outpatient antibiotic stewardship from the Centers for Disease Control and Prevention (CDC).^4^ Provider feedback based upon antibiotic prescribing, also recommended by the CDC, has demonstrated efficacy in various healthcare organizations.^5,6^ To track and provide meaningful feedback to clinicians, it is essential to have real-time and accurate data, which poses a unique challenge in the outpatient setting. Additional challenges arise outside a specific healthcare system and unified electronic medical record (EMR). Medical and pharmacy claims are a way of tracking outpatient prescriptions and have become a useful tool in these efforts, but there can be inherent limitations within the data they provide.^7,8^ To date, there have not been studies validating these claims and the accuracy of their data for ASP metrics. This study aimed to compare data extracted from Medicaid claims (MC) versus data extracted from a single academic center EMR to assess the accuracy and validity of defined stewardship metrics. Medicaid was selected for this study as an estimated 50% of children in WV are covered by Medicaid, the largest insurer of children in this region.^9^

## Methods

### Study design

Practices caring for pediatric patients within MHN, for which we could access both MC and EMR, were included. Prescribers included family medicine providers (MD, DO and advanced practice practitioners [APPs]) and pediatricians (MD and DO). Prescriber NPI numbers were used to identify outpatient pediatric MCs for the year 2022. Dental claims and non-oral antibiotics (intravenous [IV], intramuscular [IM] and topicals) were excluded. In 2022, 1,520 unique pediatric MC from these practices were identified. A randomized sample was selected for review. A power calculation indicated an optimal sample size of 307 based on a 95% confidence level and a 5% margin of error. MC and EMR data were matched for the following three categories: Medicaid member ID, date of service and provider NPI. Phantom prescriptions (those prescriptions filled in the pharmacy [having a MC] that did not have a corresponding medical visit to review in the EMR) were excluded from the analysis. A panel of 4 medical professionals conducted chart reviews (MML, JTK, JE and BH). The panel consisted of two pediatric infectious diseases specialists, one general pediatrician and one second year pediatric medical resident. Charts were equally divided among the panel. A small subset of charts was reviewed jointly to improve inter-rater reliability but was not measured systematically. Data extracted from the EMR were collected using REDCAP.

### Setting, patient population and access to data

Marshall Health Network (MHN) in Huntington, WV; provides care to patients from West Virginia (WV), Ohio (OH) and Kentucky (KY). This study included pediatric patients from 0 to 19 years of age receiving care at a MHN clinics and insured by WV Medicaid. Medicaid beneficiary eligibility, provider enrollment, pharmacy and medical claims data were provided by the West Virginia Bureau for Medical Services via a data use agreement. Data were received weekly via secure file transfer protocol (SFTP) and represented claims processed up to approximately 2 weeks prior. Patients were selected from the Cerner® EMR and matched using Medicaid member ID, date of service, and prescriber using their national provider identifier (NPI) number. All data were stored on a secure server within a restricted virtual local area network (VLAN) segment behind a firewall. This study was approved by Marshall University Institutional Review Board.

### Metrics

Antibiotic choice, duration, date of service, date of birth, diagnosis, sex and race were collected from both data sources and compared for percentage of agreement. In addition, antibiotic prescriptions were assessed for appropriateness based on billed diagnoses using a previously published classification scheme.^10^ ICD-10 diagnoses were assigned to one of the following mutually exclusive categories: appropriate, potentially appropriate and inappropriate. Examples of diagnoses appropriate for antibiotics include urinary tract infection (UTI), streptococcal pharyngitis, and bacterial pneumonia. Examples of potentially appropriate diagnoses include acute otitis media (AOM) and sinusitis. Examples of inappropriate diagnoses include viral upper respiratory tract infection and acute bronchitis. Antimicrobial selection was classified as guideline-concordant/non-concordant for infectious diagnoses with established national guidelines by the American Academy of Pediatrics and/or the Infectious Diseases Society of America (IDSA): AOM, acute sinusitis, acute pharyngitis, community-acquired pneumonia (CAP), skin and soft tissue infections (SSTI), and UTI^11–16^ (Supplemental Table 2). Outcomes of appropriateness and guideline-concordance were compared between the two data sources. Chart reviewers were blinded to claims-based classifications.

### Analysis and MC algorithms

MC data were received weekly via SFTP. The data were integrated into a secure Microsoft SQL Server within a restricted VLAN network. Diagnoses were grouped for easier identification and classification using ICD-10 Clinical Classifications Software 2024. For specific diagnosis groupings, such as UTI, additional diagnosis codes, such as “dysuria” or “painful micturition,” were manually added to the category to ensure inclusion in the algorithm. Once categorized, additional reference data were merged to assess the appropriateness of antibiotic prescribing.

When multiple diagnoses were present in MC, the algorithm prioritized “appropriate” diagnosis codes over “potentially appropriate” and “inappropriate” in relation to a given prescription and visit. For example, if a code for streptococcal pharyngitis, which always merits a prescription, appeared in the same visit as AOM, which sometimes merits a prescription, the prescription associated with that visit was evaluated based on the streptococcal pharyngitis diagnosis.

Medical claim diagnosis codes were matched to pharmacy claims using an incremental filtering algorithm, comparing diagnosis dates to prescription dates. Diagnoses were matched to prescriptions based on a 4-day period, with day 0 being the day of prescription fill, and days 1 to 3 being the preceding days. Evaluating a period of up to 7 days prior to the prescription fill resulted in a high rate of false positives, while a 3-day period produced fewer false positives and aligned with clinical reasoning, such as a Friday medical visit followed by a Monday prescription pickup (Supplemental Table 1).

When developing the code for guideline concordance, certain nuances were incorporated into the UTI group to better reflect clinician’s reasoning and intention for prescribing antibiotics. For example, though “dysuria” was not considered an appropriate diagnosis for prescribing antibiotics, for the purpose of guideline concordance, it was included in the UTI group. This approach reflects the likely clinical intent behind the antibiotic prescription, even if a UTI diagnosis was not explicitly coded. At initial visits where there was an abnormal urinalysis and suspicion of a UTI, clinicians may prescribe an antibiotic without a confirmed diagnosis (ie, positive culture) but may still code the visit as such. Therefore, the algorithms applied to the diagnosis categories and prescribing data were distinct from those assessing prescription appropriateness, taking into consideration additional factors like patient antibiotic allergies when evaluating concordance and antibiotic selection.

## Results

### Study population

**Figure 1.**
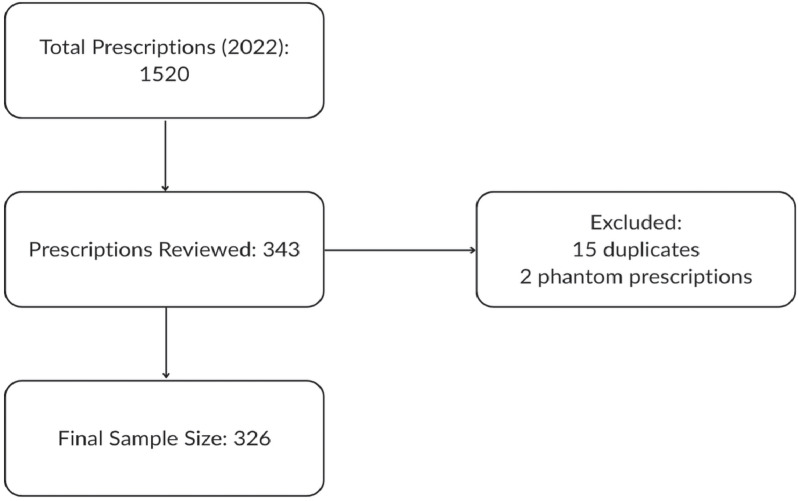
Flowchart for the selection of sample size for electronic medical record review.

A total of 60 family medicine prescribers (39 physicians, 21 APP) and 22 pediatric prescribers (all physicians) were identified within MHN that provide care to pediatric patients in the outpatient setting. We aimed for a marginally larger number than our calculated sample size to ensure this goal would be met resulting in a final randomized sample size of 326 after exclusion of duplicates and phantom prescriptions (Figure 1). Of the two MC claims identified as phantom prescriptions, only one had a recent medical visit with a complaint of ear pain but no prescribed antimicrobial at the time of that encounter. The majority (97.5%) of the prescriptions were picked up the same day of the medical visit or one day after. Only 1.4% of prescriptions were picked up after 3 days of the medical visit.

Table 1 shows the comparison of clinical and demographic data from both sources. The percent agreement between MCs and EMR was above 90% for antibiotic, date of birth, date of service, gender and state. Data elements with a percent agreement below 90% were diagnosis, duration of therapy and race. When audited, discrepancies in diagnoses were attributed to prescriptions for encounters with multiple infectious diagnoses. However, there was no discernable explanation for the discrepancies in duration. Race discrepancies were attributed to two primary factors: unknown/blank entries in the MC and instances of multiple racial identifications in the EMR.

**Table 1.** Basic data comparison: Medicaid claims vs electronic medical record data

Variable	Percent agreement (n = 326)
Antibiotic	93.60%
Date of birth	97.50%
Date of service	98.20%
Diagnosis	88.70%
Duration	83.40%
Sex	98.50%
Race	72.10%
State	97.50%

### Diagnosis appropriateness and guideline concordance

General agreement between the two data sources were approximately 90% (Table 2). When looking further into the three separate categories of diagnosis appropriateness, numbers are very similar (Table 3).

**Table 2.** Diagnosis appropriateness and guideline concordance comparison of Medicaid claims vs electronic medical record data

ASP metric	Percent agreement
Diagnosis appropriateness (n = 326)	88.7%
Guideline concordance (n = 274)	91.2%

**Table 3.** Comparison of observed frequencies by source for appropriateness in antibiotic prescribing for the visit diagnosis

Data Source	Appropriate, n (%)	Potentially appropriate, n (%)	Inappropriate, n (%)
EMR	30 (9%)	247 (76%)	41 (13%)
MC	24 (7%)	256 (79%)	46 (14%)

There were 8 prescriptions that were intended for two separate diagnoses. Our reviewers were able to categorize each separate diagnosis for appropriateness, whereas when using MCs, we were unable to reliably capture this level of granularity. A closer examination of guideline concordance shows that the percentage of agreement remains high in diagnoses with a greater number of cases, while diagnoses with fewer cases, such as CAP, pharyngitis, and UTI, have a lower agreement. For more common diagnoses, like otitis media, sinusitis, and SSTI, the percentage agreement exceeds 90% (Table 4).

**Table 4.** Agreement of Medicaid claims and electronic medical record categorization of guideline concordance per diagnosis

Diagnosis	Percent agreement
SSTI (n = 22)	95.50%
Otitis media (n = 151)	94.70%
Sinusitis (n = 78)	91.00%
Pharyngitis (n = 7)	71.40%
UTI (n = 11)	63.60%
CAP (n = 5)	60.00%
Total (n = 274)	91.20%

## Discussion

This study found that data from MCs are similar in accuracy as data from EMR for assessing the appropriateness of outpatient antibiotic prescriptions. With this work we aim to provide a solution to arguably one of the most critical obstacles outpatient ASPs face: accessing accurate, timely and reliable data to guide interventions.

Currently, most outpatient ASPs operate within existing medical systems. This is due to multiple factors including lack of infrastructure and availability of data outside of health systems. Consequently, statewide outpatient ASP efforts are limited, particularly in data-driven approaches such as provider feedback. Insurance claims offer a unique opportunity to bridge this information gap and expand inclusion beyond single healthcare systems, as most patients fill prescriptions through insurance. However, these claims are severely underutilized. The impact of this information gap becomes even more significant in rural states, like WV, where not only do high prescribing rates exist, but there are also limited unified healthcare systems. Our previous exploratory analysis indicated that Medicaid Region 4, the most rural portion of the state, is where the highest antibiotic prescription rates occurred.^17^ Yet, this area would not be reached by any of the major healthcare systems’ outpatient ASPs, as they are not located in that region. To successfully decrease the rate of unnecessary antibiotic prescriptions in WV, we need a unified data tool and to engage the most rural parts of the state.

By accessing EMR records linked to MCs, this study was able to directly compare not only the demographic information of each data source, but also, two important metrics of ASP: diagnosis and antibiotic choice of each prescription. Although discrepancies were noted, data from both sources matched ≥90% for most categories. When looking into percent agreement for diagnosis, the discrepancies were attributed to visits with multiple infectious diagnoses. From the MCs, it is not always possible to tell which diagnosis prompted the antimicrobial prescription, whereas in the EMR the reviewers could clearly identify for which diagnosis the antibiotic prescription was intended. Discrepancies in duration were harder to discern and remain unclear when audited, which may limit the reliability of using claims data alone to guide interventions focused on the duration of therapy. Race presents distinct challenges in both EMR and MC systems, highlighting the need for improvement. Both data sources show inconsistencies; for instance, not all racial categories are represented in the EMR, some patients are recorded with multiple racial identities, and certain MC entries lack race data altogether. This unreliability in racial and ethnic designation is a recognized issue, with error rates documented in pediatric healthcare systems and reported by other researchers.^18,19^ These discrepancies underscore the need for standardized methods to ensure accurate and consistent race and ethnicity data across both MCs and EMRs.

Guideline concordance proved more challenging when categorizing MCs. Ensuring a reliable algorithm that captured most codes related to the diagnoses addressed in a specific guideline was difficult. This process required subtleties as explained in the methods section for UTI. We felt it was important to assess the clinician’s antibiotic choice by the clinical intention/diagnosis they were intending to treat even if it did not exactly match the code. Based upon percent agreement, the biggest difference occurred when a single prescription was given for multiple diagnoses, where the choice may have been guideline-concordant for one diagnosis but not the other. These are cases where the MC were unable to discern if the prescription was intended for more than one diagnosis and we did not categorize that choice twice. Clinical subtleties, such as recent antimicrobial prescriptions, are missed in the MC information if only assessed singularly. For the purposes of this study in the MC portion, we did not review recent claims for the same patient, but this certainly can be incorporated into future MC-based stewardship efforts. Antibiotic allergies also don’t translate well in claims data, as clinicians rarely bill for them, potentially leading to underrecognition of appropriate second- or third-line therapy. However, these represented <1% of cases in our study. Agreement for guideline concordance for CAP, pharyngitis, and UTI was lower, likely due to a smaller sample size. Despite these limitations, key ASP targets such as inappropriate diagnoses and guideline concordances performed similarly in both data sets, supporting the utility of claims data for provider feedback.

We were expecting two problems that were not identified during this focused review: (1) phantom prescribing and (2) lack of a diagnosis in the MC. All MCs reviewed had a diagnosis present and we only identified 2 claims that did not have a paired note in EMR to review. Other studies addressing phantom prescribing report rates of about 15%,^20^ suggesting that these issues may emerge as we expand our efforts beyond MHN. MHN’s EMR requires a diagnosis as a marked field when ordering a prescription, and since almost all prescriptions reviewed were associated to a medical visit, this likely explains the presence of a diagnosis in their claims. However, when prescribers call in prescriptions, most pharmacies do not require a diagnosis. With expansion beyond MHN, particularly into rural areas of West Virginia, where provider density and healthcare access are lower, this issue may become more pronounced.

We demonstrate that MCs can provide timely and accurate data when compared to EMR. Previous efforts using MCs to target stewardship often faced a one- to two-year delay, relying on retrospective claims data .^7,21–23^ This time lag makes provider feedback less impactful, as clinicians seldom remember their actions from one or two years ago, and outdated data may not reflect their current prescribing practices. In contrast, our project leverages near real-time data. Moving forward, this timely data will allow us to deliver more relevant and impactful interventions by addressing issues closer to their occurrence, likely translating to more effective stewardship and improved patient outcomes.

The long-term impact of ASP is highly contingent on sustainability. Ultimately, effective, accurate and timely outpatient ASP interventions are needed. In the absence of a universal data source, insurance claims provide a more comprehensive database that is currently being underutilized. This study demonstrated that MC information performs similarly to EMR data; notably when assessing inappropriate prescriptions and when there is only one infectious diagnosis in that medical encounter. When accessible, insurance claims provide timely and accurate information that can reliably guide outpatient ASP interventions. Insurance providers, who have unique access to this valuable data, must become key stakeholders and partners in the challenging task of scaling outpatient stewardship interventions. Leveraging this data should drive greater commitment from insurance companies to support and expand ASP initiatives.

## Supporting information

10.1017/ash.2025.10042.sm001 Lanata et al. supplementary material Lanata et al. supplementary material

## Supplementary material

For supplementary material/s referred to in this article, please visit https://doi.org/10.1017/ash.2025.10042
