# Supplementary material for: Assessing insurance claims as a measure for outpatient antimicrobial stewardship
Source: Antimicrob Steward Healthc Epidemiol. 2025 Jun 30;5(1):e141. doi: 10.1017/ash.2025.10042 (PMC12224133; doi:10.1017/ash.2025.10042)
Supplement: Lanata et al. supplementary material [file S2732494X25100429sup001.docx]

**Supplemental Table 1**: Prescription pick up timing in relation to medical visit

|  | Day of Medical Visit | | | | | | |  |  |
| --- | --- | --- | --- | --- | --- | --- | --- | --- | --- |
| Prescription Pick up Timing | Sun | Mon | Tues | Wed | Thurs | Fri | Sat | *Total* | *Rate* |
| Same Day | 107 | 276 | 268 | 237 | 229 | 182 | 107 | *1406* | *92.50%* |
| DoS +1 | 5 | 13 | 15 | 13 | 17 | 9 | 4 | *76* | *5.00%* |
| DoS +2 |  | 2 | 5 | 4 | 2 | 1 | 2 | *16* | *1.10%* |
| DoS +3 | 7 | 4 | 4 | 2 |  | 3 | 2 | *22* | *1.40%* |
| *Daily Total Rx's* | *119* | *295* | *292* | *256* | *248* | *195* | *115* | *1520* | 100.00% |

**Supplemental Table 2**: Summary of first line therapy per national guidelines

| Diagnosis | Guideline’s Organization | First line therapy | Others/comments |
| --- | --- | --- | --- |
| UTI | AAP | Amoxicillin/clavulanate TMP/S  Sulfisaxazole  Cefixime  Cefpodoxime  Cefprozil  Cefuroxime axetil  Cephalexin |  |
| CAP | AAP and IDSA | Amoxicillin | If considering SA: clindamycin, cephalexin, or TMP/SMX  For mycoplasma and chlamydia: Azithromycin |
| GAS pharyngitis | IDSA | Pen V oral  Amoxicillin  Pen G IM | Penicillin allergy: cephalexin, cefadroxil, clindamycin, azithromycin, clarithromycin. |
| AOM | AAP | Amoxicillin high dose 80-90mg/kg/day divided BID | 2^nd^ line: amoxicillin/clavulanate Penicillin allergy: cefdinir, cefuroxime, cefpodoxime, or ceftriaxone |
| Bacterial rhinosinusitis | AAP and IDSA | Amoxicillin  Amoxicillin/clavulanate | *AAP recommends both equally, IDSA prefers amoxicillin/clavulanate over amoxicillin in children. For purposes of this study both were considered first line  Penicillin allergy: doxycycline, levofloxacin or moxifloxacin. |
| SSTI | IDSA | Purulent: TMP/SMX or Doxycycline Non-purulent: Penicillin VK, cephalexin, dicloxacillin or clindamycin |  |
